# Dispositivo de irradiación ultravioleta como herramienta pedagógica para mejorar el lavado de manos en estudiantes de enfermería

**DOI:** 10.23938/ASSN.1139

**Published:** 2025-12-04

**Authors:** Marina Gómez de Quero Córdoba, Ángel Vicario Merino, Noemí Mayoral Gonzalo, Rosa Raventós Torner, María Roser Cuesta Martínez, Jenifer Malumbres Talavera, Eva de Mingo-Fernández, Leticia Bazo Hernández

**Affiliations:** 1 Nursing Department Universitat Rovira i Virgili Vilafranca del Penedès Spain; 2 Research Group on Advanced Nursing (CARING)-161 Universitat Rovira i Virgili Spain; 3 Faculty of Health Sciences Universidad Internacional de La Rioja (UNIR) Logroño Spain; 4 Instituto de Investigación Sanitaria HM Hospitales Spain; 5 Faculty of Health Sciences University Camilo Jose Cela Madrid Spain

**Keywords:** Lavado de manos, Estudiantes de Enfermería, Ejercicio de Simulación, Radiación ultravioleta, Metodología cualitativa, Hand Disinfection, Students, Nursing, Simulation Exercise, Ultraviolet rays, Qualitative Research

## Abstract

**Fundamento:**

El objetivo es evaluar la higiene de manos en estudiantes de enfermería y conocer sus reflexiones tras una intervención pedagógica innovadora.

**Metodología:**

Estudio de simulación con estudiantes de grado de enfermería con diseño mixto: cuantitativo transversal para evaluar la efectividad de la intervención, y exploratorio cualitativo mediante escritura reflexiva. Se realizó lavado de manos con una solución hidroalcohólica que emite radiación ionizante que brilla con luz ultravioleta, empleando un dispositivo de irradiación ultravioleta para visualizar áreas limpias y sucias. Se registró el tiempo empleado y el porcentaje de superficie limpia. Tras evaluar los resultados se repitió la higiene y se compararon el tiempo empleado y la superficie limpia. Posteriormente, las personas participantes respondieron por escrito a preguntas directas y abiertas sobre el trabajo realizado.

**Resultados:**

Treinta estudiantes participaron en el estudio transversal y sesenta en el cualitativo. Las áreas de difícil limpieza fueron las yemas de los dedos y la palma de la mano, junto a los pulgares. El segundo lavado requirió 2,6 s más (p=0,012) y consiguió un 9% más de superficie mediana limpia (0,006), y las personas con limpieza ≥70% incrementaron del 36,7 al 66,7%. Las experiencias de los estudiantes mostraron grandes ventajas, como la concienciación sobre errores personales, el aprendizaje visual, y la importancia de una técnica correcta.

**Conclusiones:**

Esta estrategia educativa visual y práctica permite aumentar la concienciación del estudiantado sobre la relevancia de la higiene de manos, pudiendo contribuir a disminuir las infecciones nosocomiales provocadas iatrogénicamente.

## Abstract (en)

**Background:**

This study aims to evaluate hand hygiene performance among nursing students and to explore their reflections following an innovative educational intervention.

**Methodology:**

A simulation-based study was conducted with undergraduate nursing students using a mixed-methods design: a quantitative cross-sectional component to evaluate the effectiveness of the intervention and a qualitative exploratory component using reflective writing. Hand hygiene was performed with a hydroalcoholic solution containing ionizing agent that fluoresce under ultraviolet light. A portable ultraviolet device was used to visualize clean and missed areas. The time spent and the percentage of clean surface area were recorded. After reviewing the initial results, the handwashing procedure was repeated, and both time and clean surface area were compared. Participants then answered open-ended written questions about their experience.

**Results:**

Thirty students participated in the quantitative component and sixty in the qualitative component. The most difficult areas to clean were the fingertips, palms, and thumbs. The second handwashing attempt was 2.6 seconds longer (p=0.012), increased the median clean surface area by 9% (p=0.006), and raised the proportion of students achieving ≥70% cleanliness from 36.7% to 66.7%. Students reported notable benefits, including heightened awareness of personal errors, visual learning, and reinforced understanding of proper technique.

**Conclusions:**

This visual and hands-on educational strategy effectively increases student awareness of key aspects of hand hygiene and may contribute to reducing iatrogenic nosocomial infections.

## INTRODUCCIÓN

El lavado de manos se remonta al siglo XIX, cuando se identificó por primera vez como una medida higiénica capaz de eliminar los microorganismos de las manos. Un obstetra húngaro, Ignaz Semmelweis, descubrió la importancia de la higiene de manos en 1847, al observar que los médicos que realizaban autopsias y luego atendían partos (sin lavarse las manos) transferían partículas infecciosas que causaban fiebre puerperal en las mujeres^1^^,^^2^; el lavado de manos condujo a una reducción drástica de las muertes posparto en las madres^1^. Florence Nightingale, precursora de la enfermería moderna mediante prácticas seguras, instauró la higiene de manos obligatoria en su hospital de campaña durante la Guerra de Crimea^3^. En 2009, la Organización Mundial de la Salud (OMS) publicó una guía, *Save lives: clean your hands*, para mejorar el cumplimiento del lavado de manos en los cinco momentos en los que el personal sanitario debe aplicar esta técnica^4^^,^^5^. Y con el inicio de la pandemia COVID-19, el lavado de manos se convirtió en un mecanismo fundamental para reducir la carga viral de la enfermedad y la transmisión de las infecciones, con múltiples campañas a través de los diferentes medios disponibles para educar a la ciudadanía en la forma correcta de lavarse las manos con agua y jabón o con la solución hidroalcohólica^3^^,^^6^^,^^7^.

A pesar de su importancia y de su difusión, diversos estudios han señalado que la adherencia a la técnica de la higiene de manos es insuficiente debido a la falta de formación práctica, la escasa percepción de su relevancia y el desconocimiento sobre la correcta ejecución del procedimiento^4^^,^^6^^-^^10^.

Por ello, la higiene de manos deficiente sigue siendo uno de los principales problemas en la sanidad, debido a la elevada prevalencia de infecciones nosocomiales y resistencias bacterianas como consecuencia del uso descontrolado de antibióticos y, simultáneamente, de la práctica inadecuada/insuficiente del lavado de manos. Sin embargo y hasta la fecha, el lavado de manos sigue siendo la medida de control más sencilla, simple y económica que permite controlar y reducir la transmisión y la aparición de infecciones nosocomiales provocadas por los microorganismos patógenos institucionales^1^^,^^2^, haciendo más seguros los cuidados de salud tanto para pacientes como para sus cuidadores^11^.

En la búsqueda por optimizar la adherencia y efectividad de la higiene de manos, se han implementado soluciones tecnológicas innovadoras que superan las limitaciones de los métodos tradicionales de observación directa. Destacan los sistemas automatizados de evaluación como el uso de lámparas de luz ultravioleta combinadas con soluciones fluorescentes, tecnología inicialmente desarrollada para entornos hospitalarios que ha demostrado gran utilidad como herramienta pedagógica en la formación de personal sanitario^12^. Consiste en aplicar un gel o loción marcadora que contiene compuestos fotosensibles. Tras el protocolo de higiene de manos, la exposición a luz ultravioleta de onda corta (254 nm) revela mediante fluorescencia las zonas donde persisten residuos del marcador, indicando áreas de limpieza insuficiente (zonas interdigitales, pulgares, uñas)^12^^,^^13^.

La formación de estudiantes de enfermería en la técnica correcta de higiene de manos es fundamental para garantizar la seguridad del paciente y reducir la propagación de infecciones en el ámbito sanitario. La implementación de herramientas pedagógicas innovadoras, como los dispositivos portátiles de irradiación que utilizan luz ultravioleta para visualizar áreas de limpieza insuficiente, puede mejorar la efectividad de la formación y aumentar la concienciación de los estudiantes sobre la importancia de esta práctica^14^. Nuestra investigación busca comprender cómo las nuevas tecnologías (lámpara de luz ultravioleta y soluciones hidroalcohólicas que brillan ante esta radiación) impactan en el desarrollo de competencias prácticas, sensibilizan sobre la importancia de la higiene de manos en cuestión de infecciones nosocomiales iatrogénicas y contribuyen a la formación profesional orientada hacia una atención sanitaria controlando la seguridad de los pacientes^3^^,^^4^.

Estas ventajas, descritas por autores como Weber^4^, han impulsado su adopción en programas de simulación clínica, donde se ha observado que los estudiantes que utilizan este método desarrollan hábitos más sistemáticos y mayor conciencia de los puntos críticos descritos en la técnica de la OMS^4^^,^^5^.

El objetivo de nuestra investigación es evaluar la efectividad del uso de un dispositivo portátil de irradiación ultravioleta como herramienta pedagógica para mejorar la calidad de la higiene de manos entre estudiantes del grado en Enfermería y, además, analizar cualitativamente sus experiencias respecto a este uso.

## MATERIAL Y MÉTODOS

Se planteó un estudio mixto con estudiantes de Grado en Enfermería de la Universidad Rovira i Virgili (sede de Villafranca del Penedés) matriculados en el curso 2024-25 en la asignatura de Prácticas en Simulación II. Por un lado, se realizó un estudio descriptivo transversal para evaluar de forma preliminar la efectividad de la intervención pedagógica innovadora en un entorno controlado y, por otro, un estudio exploratorio cualitativo utilizando la escritura reflexiva para describir la experiencia vivida por las personas participantes respecto a la implementación de la combinación de ambas metodologías.

Se proporcionó información detallada sobre el estudio a todo el alumnado de Enfermería matriculado en la asignatura de Prácticas en Simulación II, asegurando que su participación no afectaría a sus calificaciones académicas ni tendría repercusiones en el curso. Se realizó un muestreo intencional, invitándoles a participar voluntariamente en el estudio. Se excluyeron aquellas personas que no habían superado la asignatura Prácticas en Simulación I.

El cálculo del tamaño muestral para la parte cuantitativa de este estudio se realizó mediante la calculadora Granmo, asumiendo una proporción esperada (p) de 0,5 que maximiza el tamaño requerido en ausencia de estudios previos, con un nivel de confianza del 95% (z = 1,96) y un margen de error (e) aceptable -dada la finalidad exploratoria y las limitaciones logísticas del estudio- entre el 10% y el 15%. La fórmula utilizada fue n=z_2_∗p∗(1−p)/e_2_, resultando un tamaño muestral de 30 participantes.

Para el estudio cualitativo se utilizaron los discursos introducidos por los estudiantes en un cuestionario Google forms donde se realizaron preguntas abiertas.

Todos los participantes firmaron un consentimiento informado, tras recibir información detallada sobre los objetivos, procedimientos, riesgos y beneficios del estudio. El comité ético de la Universidad Camilo José Cela emitió dictamen favorable (CAS 6763-0) a la intervención que iba a realizarse en estudiantes, que también recibió la autorización del Comité Ético de Investigación en Personas, Sociedad y Medio Ambiente de la Universidad Rovira i Virgili (CEIPSA-2023-PRD-0031).

### Proceso de intervención

La intervención consistió en una sesión de trabajo en grupo de dos horas de duración, con ocho a diez estudiantes por grupo. Los participantes trabajaron con la metodología en simulación.

**Figura 1:**
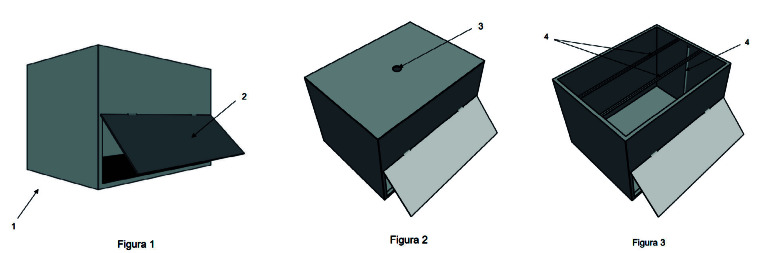
Dispositivo portátil de irradiación con luz ultravioleta. El dispositivo (1) fue patentado en el año 2022 por este equipo investigador. Las manos se introducen dentro del dispositivo por una trampilla batiente (2). La fuente de luz ultravioleta (4) permite detectar la radiación no ionizante del gel hidroalcohólico. Una pequeña apertura en la parte superior de la caja (3) permite obtener imágenes.

**Figura 2:**
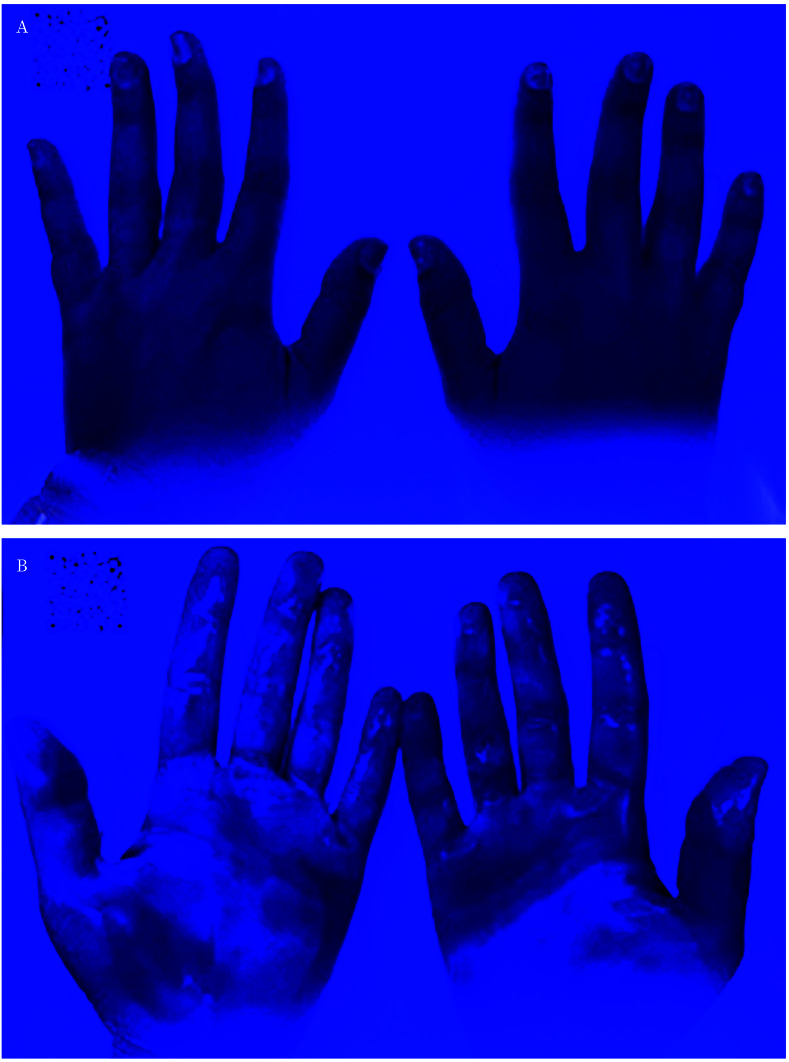
Fotografía tras aplicar la solución ionizante. Se visualizan las zonas sucias de las manos (oscuras) y limpias (brillantes), lo que permite evaluarlas. **A**. Manos tras realización deficiente del lavado de manos. **B**. Manos tras higiene realizada más correctamente.

1. Responder al cuestionario accediendo a través del smartphone al código QR proporcionado.
2. Realizar su higiene de manos aplicando dos dosis de solución hidroalcohólica Sterilium© con reactivo Visirub© (solución que, donde persiste, emite radiación ionizante capaz de inducir brillo con la luz ultravioleta). Se cronometró el tiempo en segundos empleado en el lavado de manos y se registró.
3. Evaluar la calidad del lavado colocando ambas manos dentro del dispositivo portátil de irradiación con luz ultravioleta para detectar radiaciones no ionizantes (Fig. 1). Se fotografiaron ambas palmas y ambos dorsos de cada participante con las cámaras de los smartphones de los investigadores (Fig. 2). Las fotografías se almacenaron en orden según el número de la muestra y se analizaron con el programa Photoshop 2022.

Para calcular el área correctamente lavada se siguieron estos pasos:

- se selecciona la imagen del dorso de la mano y se abre;
- se determina la medida a utilizar. En el caso de las imágenes tomadas, el código QR tiene una medida de 1,5 cm, por lo que se hace corresponder esa longitud con los píxeles correspondientes;
- con la herramienta lazo se selecciona toda la mano para calcular el área, y se registra este valor;
- con la herramienta lazo se selecciona la parte limpia de la mano (brilla bajo la luz UV) que proporcionará el área seleccionada, y se registra el valor;
- se calcula la diferencia entre estos valores, cuyo resultado es el área de la mano que ha sido correctamente lavada con el gel de solución hidroalcohólica;
- se repite el proceso con la fotografía de la otra mano;
- se repite el proceso con la palma de cada mano.

4. Verbalizar los errores cometidos en la higiene de manos durante el debrifing.
5. El proceso de higiene de manos y su análisis cuantitativo se repitió tras el debrifing (90 minutos entre ambos procedimientos) para analizar la presencia de diferencias.

Al finalizar la actividad, los estudiantes reflexionaron sobre las ventajas e inconvenientes experimentados con la combinación de dichas metodologías activas, y escribieron online notas reflexivas que registraron como respuesta a preguntas abiertas.

### Análisis estadístico

Para el análisis cuantitativo se utilizó el programa Jamovi, basado en el motor de análisis estadístico R. Las variables categóricas se describieron mediante frecuencias y porcentajes, y las variables cuantitativas, tras comprobación de normalidad con Shapiro-Wilk, mediante media y desviación estándar (DE), o mediana y rango intercuartílico (RIC). Las comparaciones entre grupos se efectuaron mediante las pruebas Chi-cuadrado y t de Student o U de Mann-Whitney, y la asociación entre variables cuantitativas se analizó mediante correlación de Spearman (ρ). Las comparaciones pre-post actividad dentro del grupo de estudiantes se analizaron con las pruebas t de Student pareada o Wilcoxon.

Para el análisis cualitativo, se realizó un análisis temático que consistió en identificar el contenido más descriptivo para obtener códigos y, posteriormente, reducir e identificar los grupos (categorías) más codificados. Este proceso se realizó por separado en cada nota reflexiva. Después se llevaron a cabo reuniones conjuntas para combinar los resultados del análisis y representar la perspectiva de los participantes. En los casos de posibles discrepancias, la identificación del tema se basó en el establecimiento de un consenso entre miembros del equipo de investigación. La saturación fue evidente en el análisis, ya que los datos se repitieron y no se proporcionó información nueva. No se utilizó un software cualitativo para analizar los datos.

## RESULTADOS

La muestra incluyó 30 estudiantes en la parte cuantitativa y 60 estudiantes en la parte cualitativa, lo que supuso un 95,75% de participación sobre el total de matriculados.

### Estudio cuantitativo

La muestra obtenida estaba compuesta por 30 estudiantes, el 78% mujeres, con edad media 22 años (rango: 18 a 26). El 68% de participantes afirmó haber recibido algún tipo de formación sobre las técnicas de lavado de manos durante el último año, y el 80% declaró que utiliza solución hidroalcohólica con regularidad.

El tiempo empleado en la higiene de manos siguió una distribución normal (19,7 segundos; DE=6,4), mientras que la superficie limpia no la siguió (mediana=61; RIC: 55-70). Las zonas de la mano que más frecuentemente no se limpiaron correctamente fueron las yemas de los dedos en el 85% de las manos, el centro de la palma en el 82%, y el pulgar completo en el 75% (Fig. 2).

El 63,3% de estudiantes lograron un porcentaje moderado de área limpia de la mano, entre el 60% y el 70%, mientras que el 26,7% obtuvo porcentajes inferiores y el 10% superiores (Tabla 1). Se observa dispersión de los datos de efectividad del lavado de manos dentro del grupo estudiado.

Durante la segunda higiene de manos aumentaron tanto el tiempo medio (22,3 segundos; DE=5,9) como el porcentaje mediano de superficie limpia conseguida (70%; RIC: 63-78). Estos resultados estadísticamente significativos indican que los estudiantes dedicaron más tiempo a la higiene (2,6 segundos; p=0,012) y obtuvieron un 9% más de superficie limpia (p=0,006).

El porcentaje de estudiantes que lograron una limpieza ≥70 % se incrementó en un 82% (66,7% frente a 36,7%), indicando una mejora significativa y notable en la eficacia de la técnica tras la intervención pedagógica.

La relación entre el tiempo dedicado a la higiene en ambos procesos de higiene y el porcentaje de superficie limpia conseguida muestra una correlación positiva moderada y estadísticamente significativa (ρ de Spearman =0,46; p=0,011).

**Tabla 1:** Resultados de la higiene de manos pre- y post-actividad con el dispositivo de luz ultravioleta en un grupo de estudiantes de enfermería

Variables	Pre	Post	p*
Tiempo (segundos), media (DE)	19,7 (6,4)	22,3 (5,9)	0,012
Superficie limpia (%), mediana (RIC)	61 (55-70)	70 (63-78)	0,006
40%	4 (13,3)	0	0,018
50%	4 (13,3)	2 (6,7)	
60%	11 (36,7)	8 (26,7)	
70%	8 (26,7)	12 (40)	
80%	2 (6,7)	6 (20)	
90%	1 (3,3)	2 (6,7)	

*: valores de p correspondientes a t de Student pareada para comparación de medias, Wilcoxon para comparación de medianas, y X^2^ para comparación de porcentajes; DE: desviación estándar; RIC: rango intercuartílico.

### Estudio cualitativo

*Ventajas*

De las reflexiones proporcionadas por los estudiantes respecto a las ventajas de la metodología de simulación en higiene de manos surgieron distintas categorías:

Desarrollo cognitivo y concienciación

Los estudiantes indicaron que el uso de estas metodologías combinadas les ayuda a afianzar los conceptos (E14: “Afianza conceptos”), les ayuda a realizar una representación visual y práctica de la teoría, (E5: ”Permite asentar conocimientos de manera visual”), mejora la comprensión de conceptos complejos (E9: “El aprendizaje visual conciencia de una manera mayor”: E15: “Es una manera de aprender detectando tus errores”) y fomenta el pensamiento crítico o alternativo (E22: “Se asimilan conceptos de otra manera”; E23: “Cuando ves las zonas de las manos que no has lavado, te conciencias”).

Uso de luz ultravioleta

Los estudiantes reconocieron que potencia el aprendizaje en la visualización del error (E6: “Con el producto y la luz ultravioleta puedes ver rápidamente zonas de la mano sucias y limpias” y E19: “Es una dinámica que permite detectar zonas de la mano no limpias rápido”; E10: “Nos ha servido para poner en práctica de manera útil los conceptos aprendidos en teoría” E44: “Después de este taller veo que es difícil hacerlo correctamente sin dejarte zonas de la manos sin lavar”), lo identifican como una exploración de formas original de aprendizaje (E60: “Permite explorar otras formas de aprender“; E38: “Piensas y te das cuenta de que nos hemos lavado las manos mal todo este tiempo, pensando que lo hacíamos bien“) y permite la puesta en marcha sistemática del lavado de manos siguiendo las normas de la OMS (E31: “Te hace pensar de una forma diferente; E54: Tenemos el póster con los pasos a seguir de la higiene de manos y pensábamos que lo hacíamos bien, y no lo hacemos”).

Los estudiantes valoran positivamente el carácter práctico y dinámico de la metodología (E50: “Es más práctico y dinámico“; E61: “Muy interactivo y participativo“; E27: “Es una forma más dinámica de aprender, dándote cuenta de los errores“), además reconocen una mayor implicación en la actividad gracias a la metodología (E46: “No es el típico método teórico, es algo diferente, lo ves y te lo crees”. E9: “aprendes a no equivocarte”).

Emociones que genera la combinación de teoría y práctica

El taller de simulación fue calificado como ameno, dinámico, proactivo y útil (E56: “Es entretenido y útil; E12: “Hizo la clase más amena; E6: “Fue diferente a una clase tradicional ya que tener el dispositivo y la luz ultravioleta nos hizo darnos cuenta de las zonas de las manos sucias). El carácter interactivo de la clase fue identificado como una motivación adicional (E32: “Es más visual).

Los estudiantes identifican que la actividad fomenta la seguridad del paciente pudiendo reducir infecciones nosocomiales (E15: “Nos hemos concienciado más sobre las zonas de la mano sucias, hemos aprendido y hemos profundizado sobre el tema”), mejora en la resolución de problemas a la hora de memorizar los pasos de la higiene de manos (E27: “Creo que esta metodología aparte de reducir la suciedad en las manos, nos motiva más a ser más cuidadosos en la higiene de manos con nuestros pacientes” y crea un ambiente de seguridad del paciente (E6: “Me ha permitido conectar directamente con la actividad y con las personas que en el futuro cuidaré, focalizando en el orden a seguir en la higiene de manos, sabemos las zonas que más fácilmente se quedan sucias aun lavándote las manos, esto nos permitirá incidir en esas zonas”).

Concentración

Se reconoció que mejora la concentración, generando un mayor enfoque en la actividad (E21: “Te enfocas en la actividad y te olvidas de lo demás”), produciendo una reducción de distracciones externas (E13: “Ayuda a centrarte en el momento, visualizando en directo”) y evasión del entorno habitual (E11: “Es una actividad que te abstrae”).

*Inconvenientes*

Las personas participantes encontraron dificultades para exponer ideas propias en relación a la higiene de manos. También mostraron desconocimiento sobre cómo replicar la higiene de manos en un ambiente no controlado como el contexto hospitalario (E33: “Dificultad para realizar los pasos correctos de la higiene de manos cuando esté en el hospital”; E3: “Dificultad para hacer higiene de manos entre paciente y paciente”; E19: ”Difícil llevarlo al entorno hospitalario y que salga el lavado de manos perfecto”).

## DISCUSIÓN

Los resultados obtenidos en este estudio muestran diferencias significativas en la efectividad de la higiene de manos antes y después de la actividad con el dispositivo de luz ultravioleta. Tras la actividad, los participantes mostraron mayor dedicación al procedimiento y lo realizaron con mayor eficiencia. Esto sugiere que dedicar mayor tiempo al lavado se asocia con una mayor limpieza de la superficie de la mano, y confirma que se ha generado un aprendizaje profundo y significativo de la seguridad del paciente ligado a la higiene de manos.

Estos resultados coinciden con los de un ensayo clínico aleatorizado que también utilizó una lámpara de luz ultravioleta y que observó que su uso mejoró significativamente la adherencia de estudiantes de medicina a las recomendaciones de higiene de manos establecidas por la OMS^6^. Estas observaciones refuerzan la hipótesis de que ofrecer retroalimentación visual inmediata y concreta contribuye a potenciar conductas correctas y a sensibilizar respecto a las consecuencias derivadas de una praxis deficiente de higiene de manos.

Si bien desde los años 1920 se reconocen los beneficios del lavado de manos para la prevención de infecciones nosocomiales, estudios recientes confirman que su correcta ejecución sigue representando un reto, incluso para profesionales sanitarios entrenados. Tal como lo evidencian investigaciones anteriores, persisten zonas frecuentemente olvidadas durante el lavado, como los pulgares, espacios interdigitales, nudillos, uñas y muñecas, lo que subraya la necesidad de reforzar estas prácticas mediante estrategias innovadoras de enseñanza^7^^,^^8^.

Se evidencia que, aunque el lavado de manos es una práctica cotidiana y aparentemente sencilla, su correcta ejecución requiere atención y técnica y aun así no siempre se consigue en totalidad^3^^,^^4^^,^^9^^,^^10^. Esto refuerza la necesidad de implementar estrategias educativas que no solo enseñen los pasos correctos, sino que también conciencien sobre los riesgos asociados a una higiene deficiente en entornos hospitalarios para reducir las infecciones nosocomiales ya que muchas veces son iatrogénicas.

Nuestro estudio coincide con la literatura en las zonas más frecuentemente descuidadas en el lavado de manos ya fueron estudiados en el año 2015 exponiendo que los pulgares, espacios interdigitales, nudillos, uñas y muñecas^2^^,^^5^^,^^10^^,^^11^.

Sin embargo, resulta novedoso abordar la concienciación combinándolo con un instrumento que hace al estudiante ver sus errores en vivo, especialmente cuando se implementa en talleres de simulación que buscan impactar de manera notable en la formación y sensibilización de futuros profesionales sanitarios.

Nuestra investigación está enfocada en las experiencias del estudiantado, al igual que otros estudios^2^^,^^5^^,^^11^. Mediante el estudio cualitativo pudimos profundizar en las experiencias individuales al combinar la metodología en simulación con el poder disponer del dispositivo portátil con exposición a luz ultravioleta, lo que ha permitido que el alumnado obtuviese una mayor concienciación. Subrayamos la importancia de combinar técnicas tradicionales de aprendizaje con metodologías innovadoras (simulación, TICs) para mejorar la calidad del lavado de manos. En nuestro estudio se incrementó la concienciación y también el tiempo dedicado al lavado de manos, resultados que coinciden con los de Fernandez-Prada^18^ tras un taller de lavado de manos impartido por sanitarios entrenados.

Por otra parte, los talleres de simulación ofrecen un espacio seguro y controlado para practicar y corregir errores sin consecuencias negativas inmediatas^4^. Esto fomenta un aprendizaje activo y participativo que puede ser más efectivo que las metodologías tradicionales basadas únicamente en teoría y, en el caso del lavado de manos, se puede vincular a diferentes procedimientos clínicos que requieren realizar una correcta higiene de manos por la seguridad del paciente^10^^-^^13^.

Como línea de investigación futura, se podrían incorporar aspectos con un enfoque similar al de Okuroğlu y col que demostró la eficacia del entrenamiento asistido por video y la retroalimentación visual usando tecnología UV en estudiantes de enfermería^14^. Este ensayo clínico aleatorizado encontró que el cumplimiento en higiene de manos fue significativamente mayor en estudiantes expuestos a estas herramientas que en el control (52,6 vs. 39,1%), así como mejores puntuaciones en listas de habilidades prácticas. La combinación de entrenamiento visual, retroalimentación inmediata y recursos multimedia fortaleció tanto las creencias como las prácticas respecto a la higiene, subrayando el valor de estrategias educativas innovadoras en la formación sanitaria.

El impacto pedagógico de visualizar las áreas mal lavadas mediante luz ultravioleta puede ser el factor clave de nuestra investigación en cuanto al cambio de comportamiento ya que la totalidad de nuestros participantes lo mencionan en los resultados. Aunque otros autores han estudiado el efecto del aprendizaje en este ámbito^2^^,^^5^^,^^11^, nuestro estudio es novedoso al mostrar los resultados de la higiene de manos en directo con la luz ultravioleta. Los participantes aprenden a mejorar su técnica, y también desarrollan mayor responsabilidad hacia su propia higiene y hacia la prevención de infecciones^11^^,^^12^. Este aspecto es especialmente relevante en contextos sanitarios, donde el lavado de manos adecuado es crucial para garantizar la seguridad del paciente y del propio personal sanitario. Esta iniciativa tiene un recorrido largo; los datos obtenidos sugieren que este enfoque podría replicarse en contextos educativos (comedores escolares, por ejemplo) o en instituciones sanitarias donde la higiene de manos es fundamental^2^^,^^10^^,^^11^, incluso en otros contextos (fábricas de alimentación, restauración, etc.), podría incrementar el compromiso con la correcta higiene de manos.

Este estudio no está exento de limitaciones. En primer lugar, el tamaño muestral reducido en el estudio cualitativo limita la robustez y generalización de los resultados, por lo que deben interpretarse con cautela. Además, al tratarse de reflexiones escritas inmediatamente después de una actividad educativa -y en algunos casos en entornos percibidos como evaluativos-, es probable que las respuestas estén influidas por el deseo de ofrecer una imagen positiva ante el profesorado, introduciendo un posible sesgo de deseabilidad social. Para futuros estudios, sería conveniente garantizar un mayor anonimato o utilizar entrevistas conducidas por investigadores externos con el fin de minimizar este sesgo. En cuanto al dispositivo portátil de irradiación con luz ultravioleta, aunque su utilidad pedagógica ha sido evidente, no se han discutido suficientemente sus posibles limitaciones técnicas. Aspectos como la precisión en la detección de residuos, la necesidad de calibración periódica, la variabilidad de resultados según la iluminación ambiental o el posicionamiento de las manos, así como la posible variabilidad interobservador en la interpretación de las imágenes, podrían influir en la fiabilidad de los resultados. Por tanto, se recomienda estandarizar los procedimientos de obtención y análisis de las imágenes, así como analizar la concordancia entre observadores (por ejemplo, mediante coeficiente kappa) para reforzar la objetividad de las evaluaciones. Por último, el diseño transversal no permite evaluar la sostenibilidad de los cambios observados en la técnica del lavado de manos y en la concienciación respecto al mismo. Sería necesario implementar estudios longitudinales que analicen la retención del aprendizaje y el impacto sostenido de este tipo de intervenciones a medio y largo plazo.

En conclusión, la combinación de técnicas tradicionales con herramientas innovadoras (como el dispositivo portátil de luz ultravioleta) en talleres prácticos de simulación fue eficaz para mejorar la concienciación, el pensamiento crítico y la comprensión práctica de la higiene de manos en estudiantes de enfermería. Esta metodología no solo facilita la detección de errores en tiempo real y refuerza la cultura de seguridad del paciente, sino que también genera experiencias de aprendizaje dinámicas y motivadoras.

Futuras líneas de investigación podrían enfocarse en realizar un seguimiento longitudinal que permita evaluar el impacto sostenido de la intervención a lo largo del tiempo. Esto nos permitirá comprender mejor la efectividad real y la durabilidad de las intervenciones, e identificar factores relacionados con la continuidad y los beneficios para las personas participantes.

## Data Availability

Se encuentran disponibles bajo petición a la autora de correspondencia.
